# Supplementary material for: Identification of core therapeutic targets for Monkeypox virus and repurposing potential of drugs: A WEB prediction approach
Source: PLoS One. 2024 Dec 6;19(12):e0303501. doi: 10.1371/journal.pone.0303501 (PMC11623562; doi:10.1371/journal.pone.0303501)
Supplement: S1 Table — (DOCX) [file pone.0303501.s001.docx]

Table s1 Physicochemical properties of 12 potential target protein sequences and sequence similarity to human and orthopoxvirus

|  | A49R | H1L | C7L | D6L | A22R | C19L | C3L | E4R | A41L | E13L | I7L | A50R |
| --- | --- | --- | --- | --- | --- | --- | --- | --- | --- | --- | --- | --- |
| Weight | 23289.7 | 19727.8 | 25705.3 | 14337.6 | 49147.1 | 41808.7 | 4982.7 | 25076.8 | 25371.9 | 61937.0 | 79605.2 | 63574.3 |
| PI | 5.3 | 9.2 | 4.7 | 6.5 | 5.6 | 6.5 | 4.3 | 7.0 | 5.0 | 5.2 | 7.5 | 7.5 |
| Extinction | 28420 | 17880 | 28310 | 18450 | 49280 | 52370 | 8940 | 45380 | 24870 | 58680 | 72660 | 58790 |
| Instability | 50.4 | 46.8 | 41.4 | 29.0 | 36.3 | 29.8 | 29.7 | 42.1 | 39.4 | 28.8 | 30.0 | 32.2 |
| Aliphatic | 90.8 | 86.0 | 84.1 | 82.7 | 92.8 | 93.8 | 95.3 | 92.1 | 81.9 | 90.1 | 86.4 | 90.0 |
| Hydrophathicity | -0.245 | -0.308 | -0.248 | -0.247 | -0.258 | -0.112 | 0.160 | -0.258 | -0.360 | -0.200 | -0.236 | -0.344 |
| Human Identity | 42.8 | 26.9 | <10% | 94.3 | <10% | 16.6 | 24.2 | <10% | <10% | 26.7 | 19.6 | 56.6 |
| VAR Identity | 98.5 | 98.8 | 83.6 | 92.1 | 96.7 | 97.3 | 78.6 | 96.8 | 91.4 | 98.9 | 98.8 | 96.4 |
| VAC Identity | 99.0 | 100.0 | 83.6 | \ | 97.4 | 98.7 | 95.2 | 98.6 | 94.1 | 98.9 | 99.1 | 97.8 |
| CPV Identity | 99.0 | 98.8 | 87.2 | 79.7 | \ | 98.7 | 97.6 | \ | 94.1 | \ | \ | 97.8 |
