# Supplementary figures and images for: Identification of core therapeutic targets for Monkeypox virus and repurposing potential of drugs: A WEB prediction approach
Source: PLoS One. 2024 Dec 6;19(12):e0303501. doi: 10.1371/journal.pone.0303501 (PMC11623562; doi:10.1371/journal.pone.0303501)

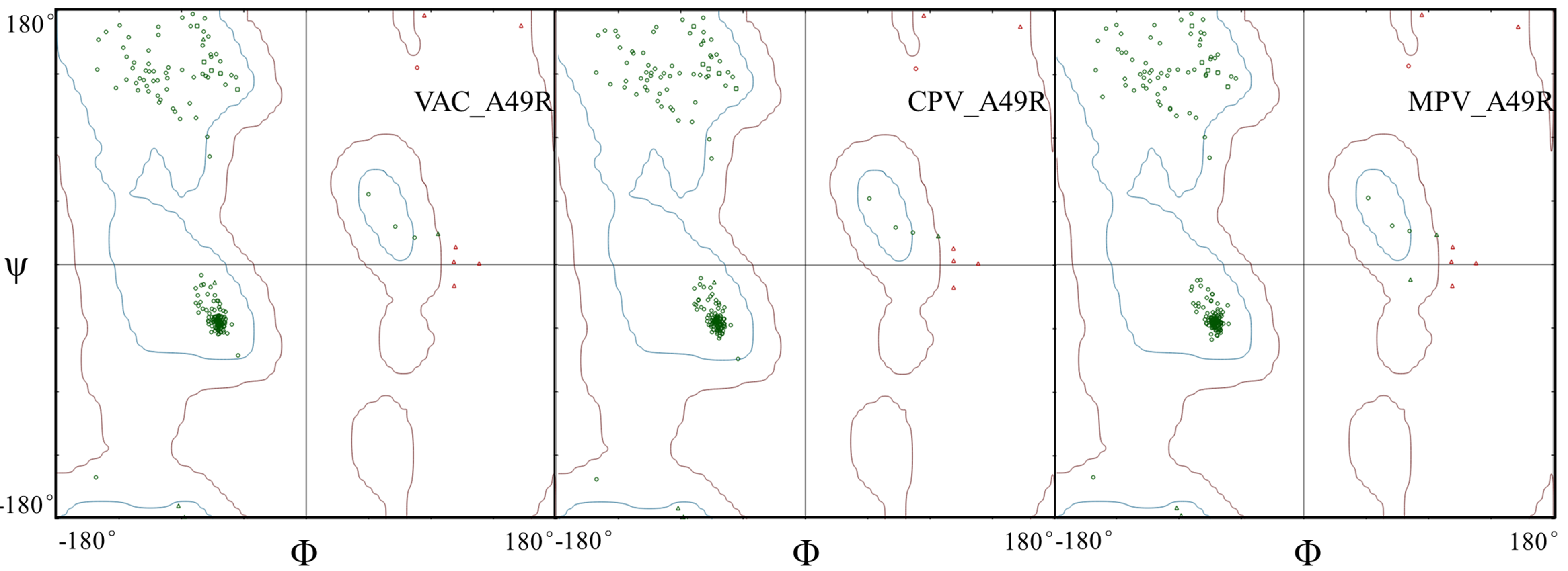

Supplement: S1 Fig — (TIF) [file pone.0303501.s003.tif]

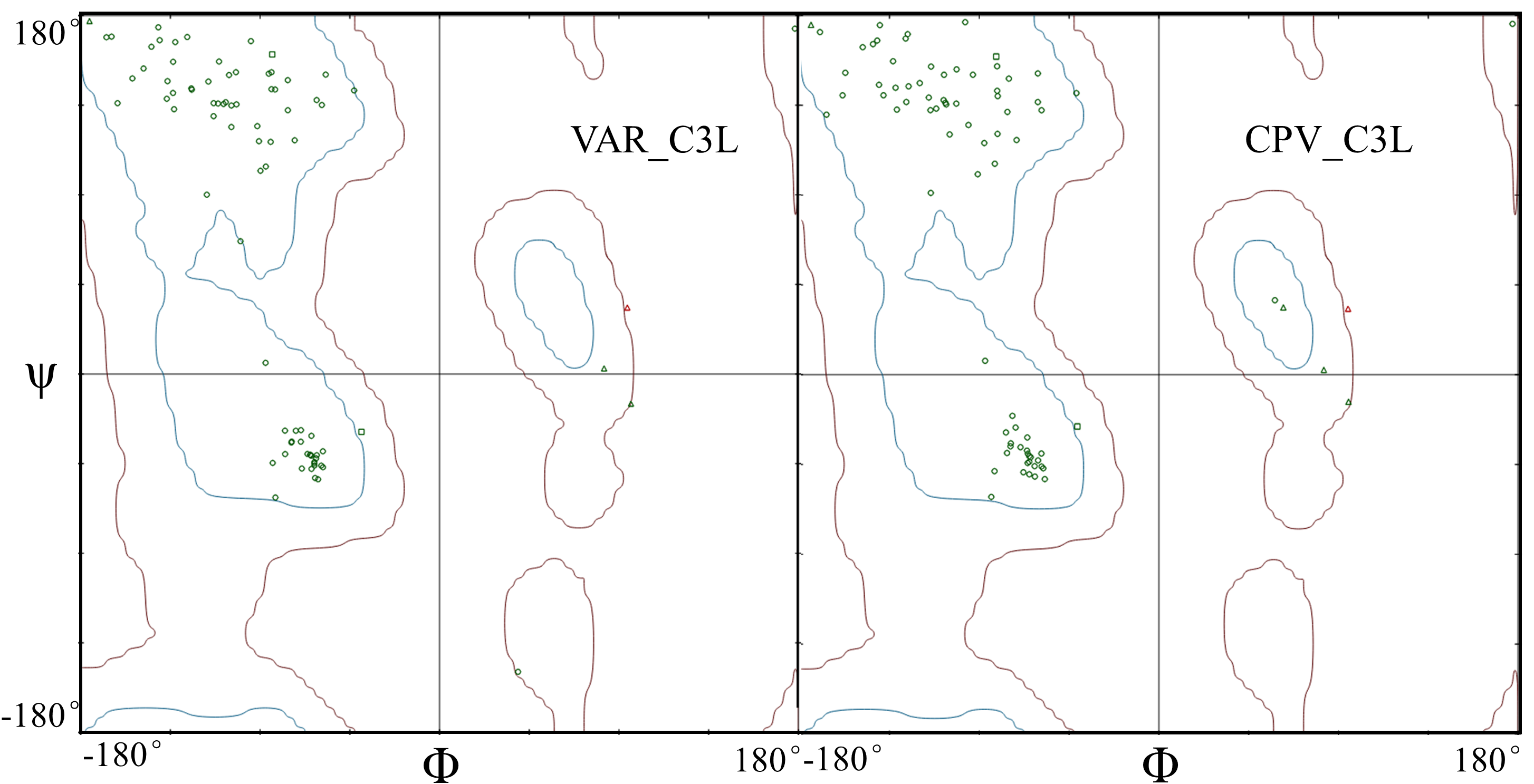

Supplement: S2 Fig — (TIF) [file pone.0303501.s004.tif]

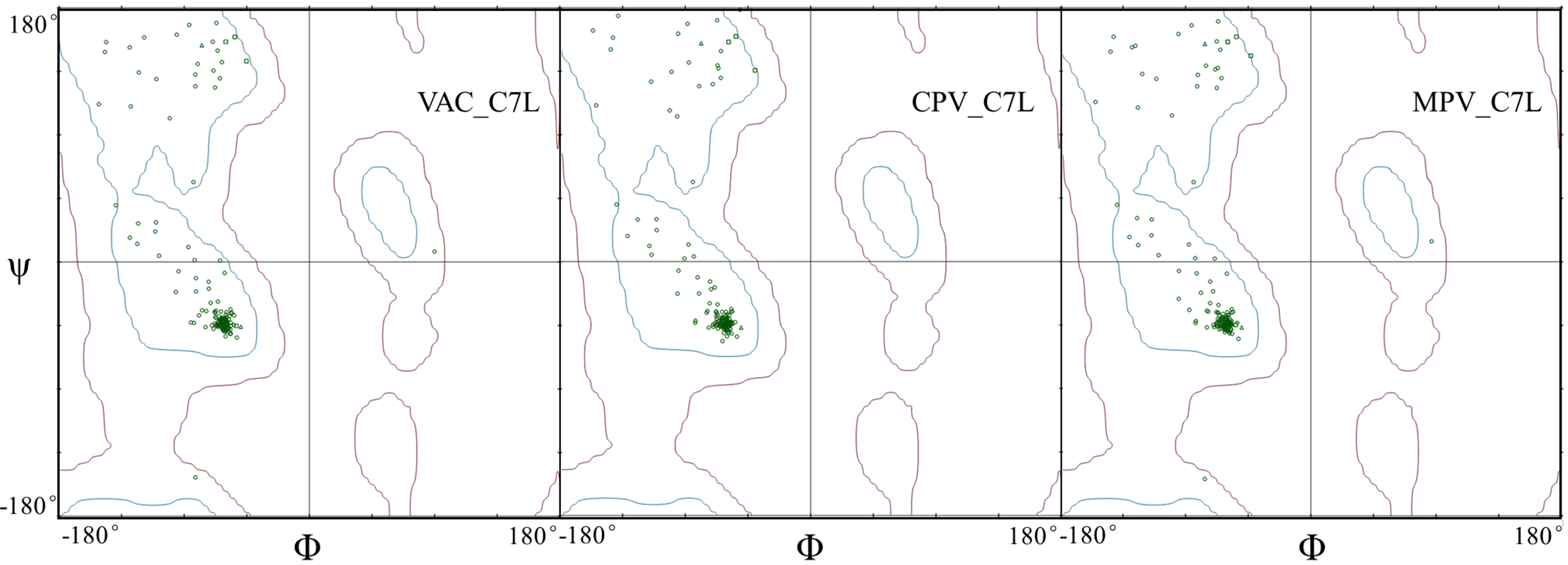

Supplement: S3 Fig — (TIF) [file pone.0303501.s005.tif]

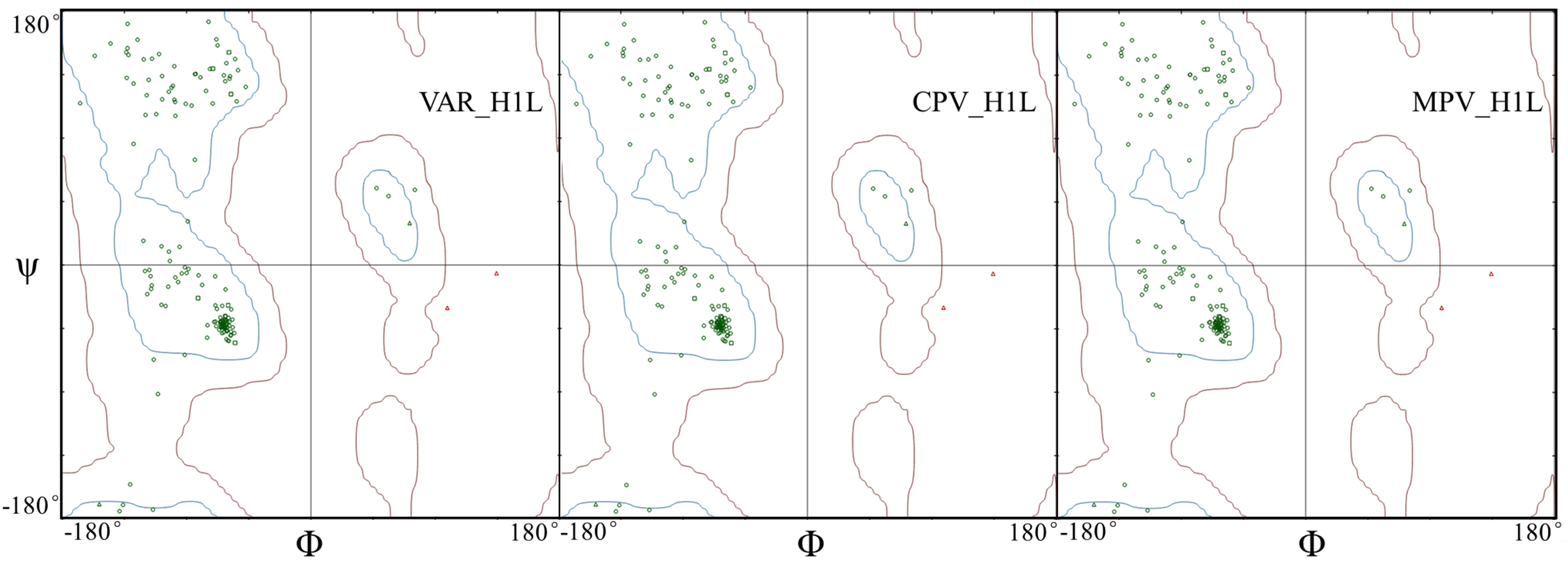

Supplement: S4 Fig — (TIF) [file pone.0303501.s006.tif]

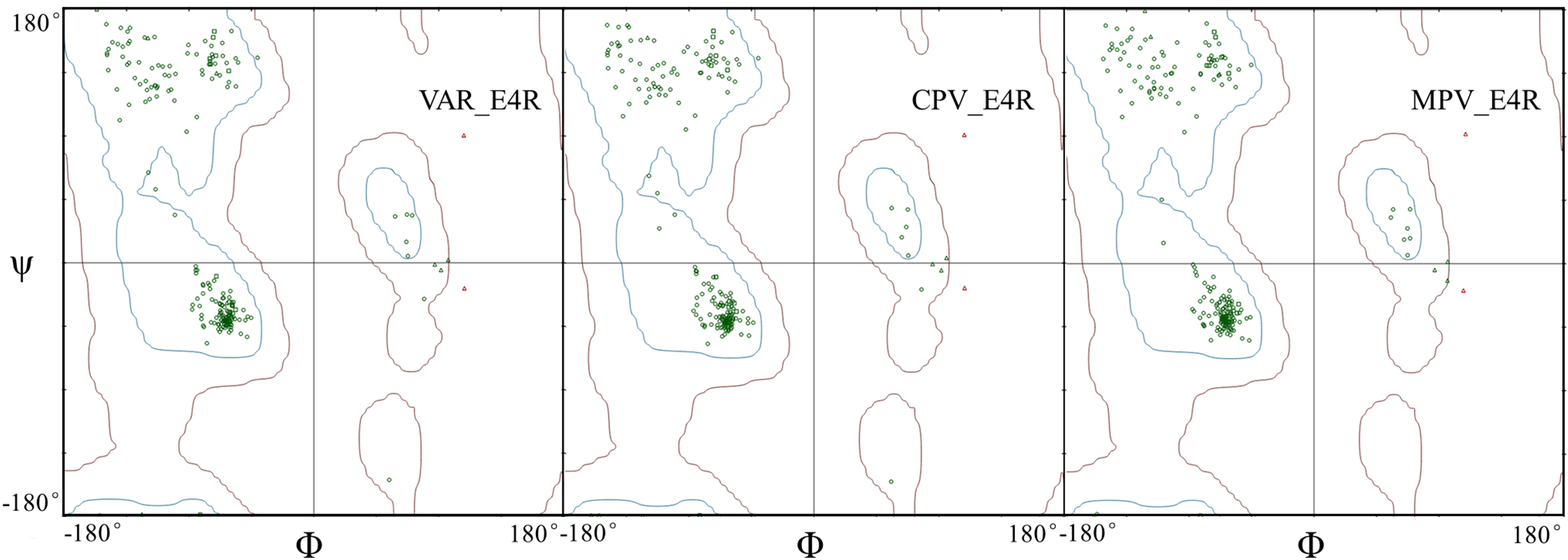

Supplement: S5 Fig — (TIF) [file pone.0303501.s007.tif]

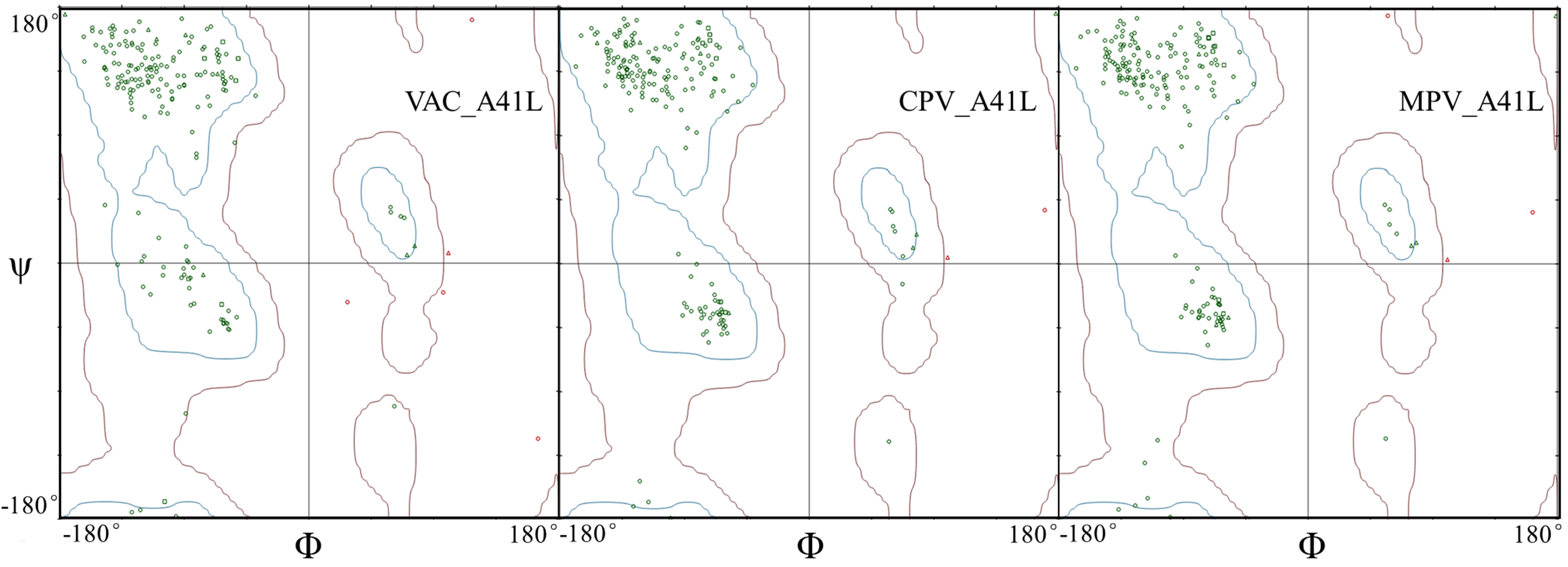

Supplement: S6 Fig — (TIF) [file pone.0303501.s008.tif]

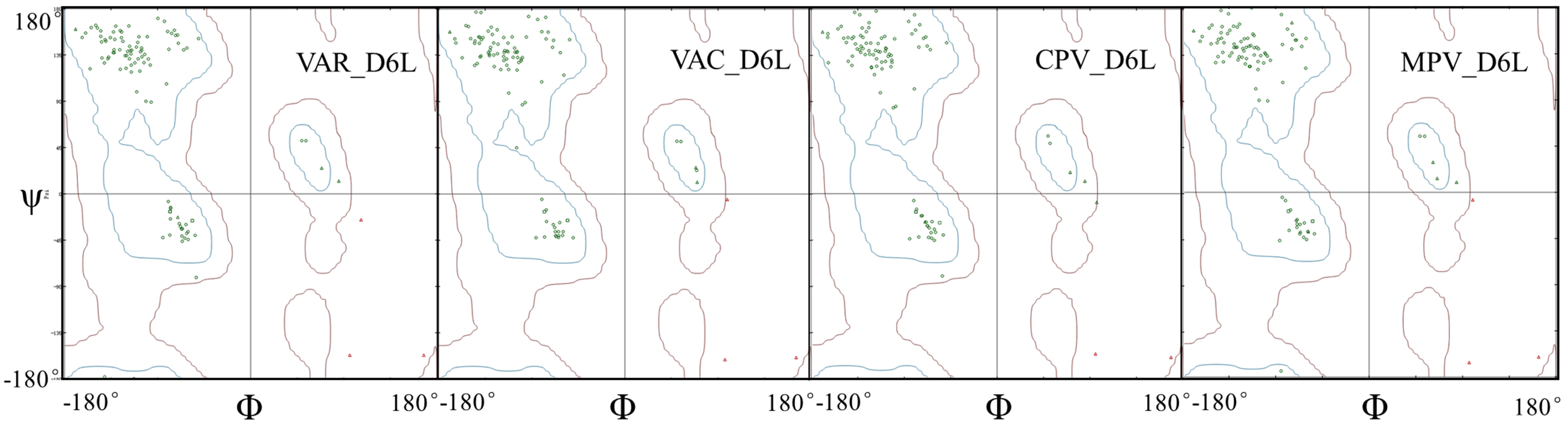

Supplement: S7 Fig — (TIF) [file pone.0303501.s009.tif]

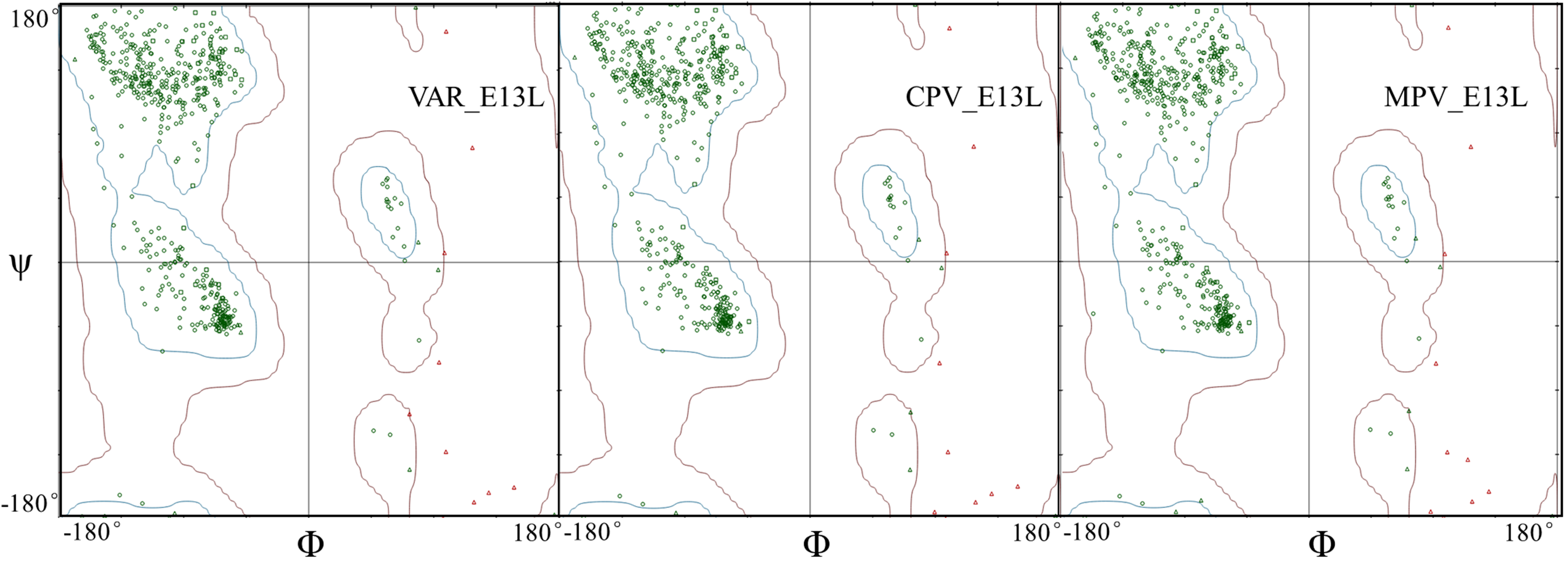

Supplement: S8 Fig — (TIF) [file pone.0303501.s010.tif]

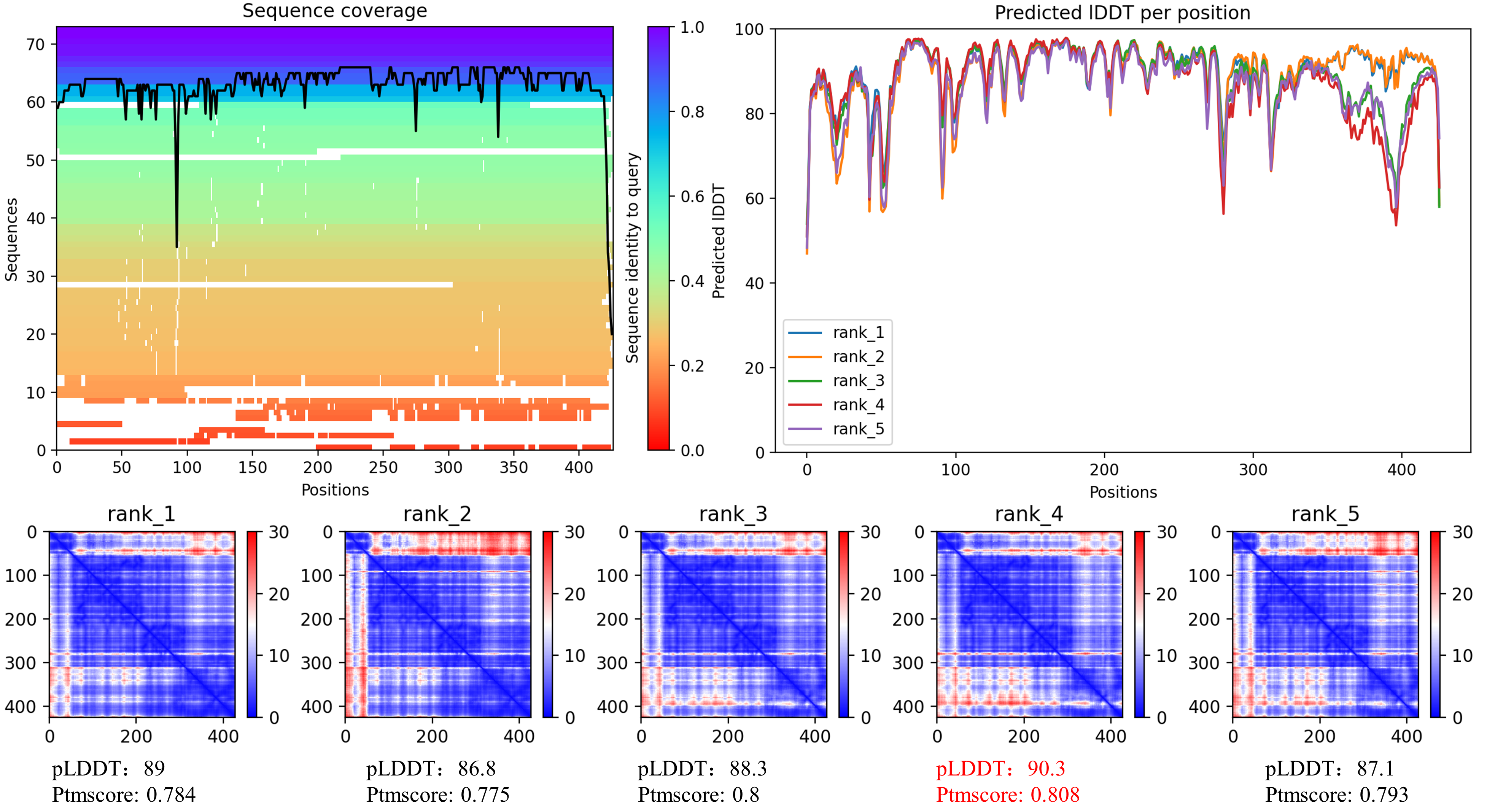

Supplement: S9 Fig — (TIF) [file pone.0303501.s011.tif]

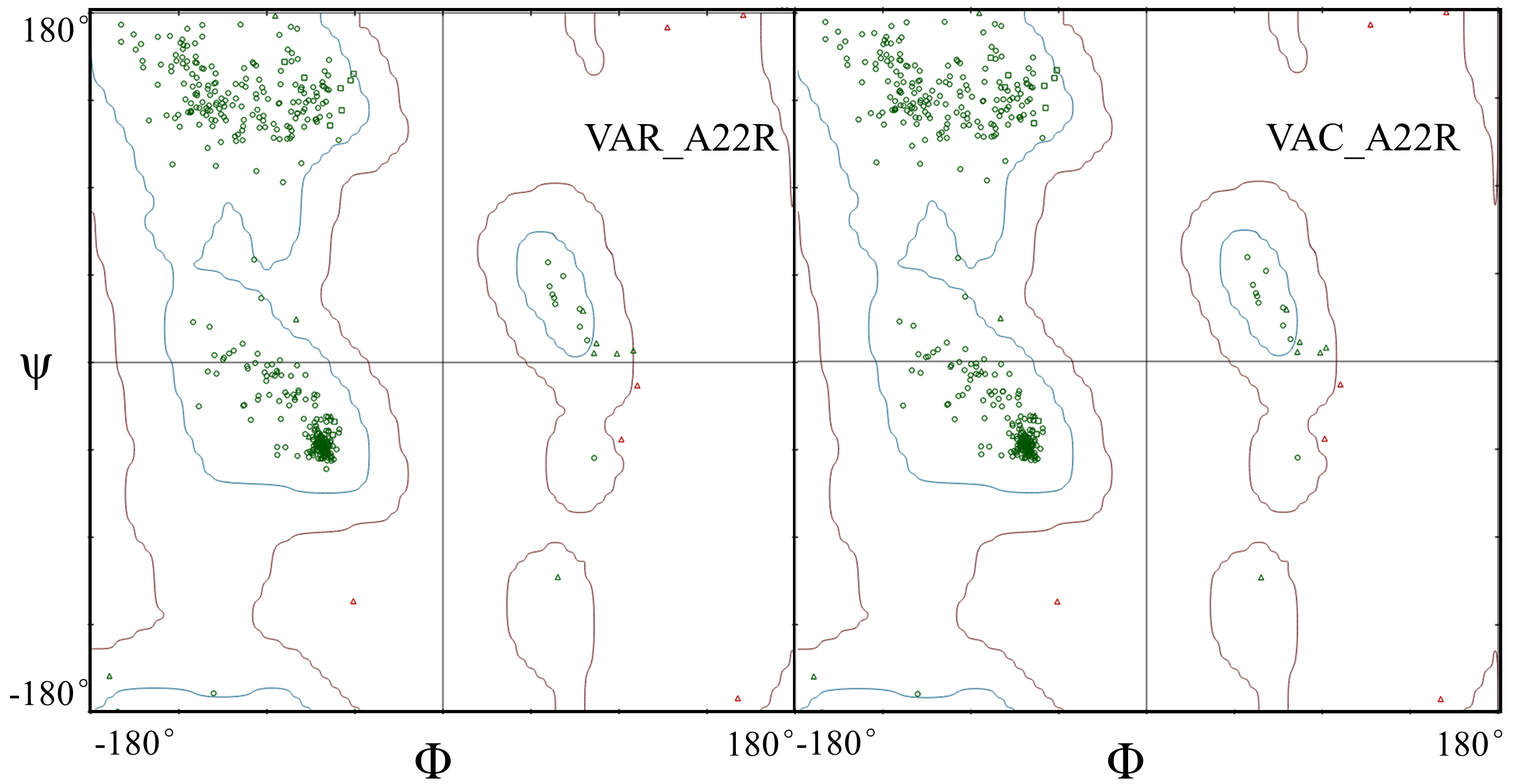

Supplement: S10 Fig — (TIF) [file pone.0303501.s012.tif]

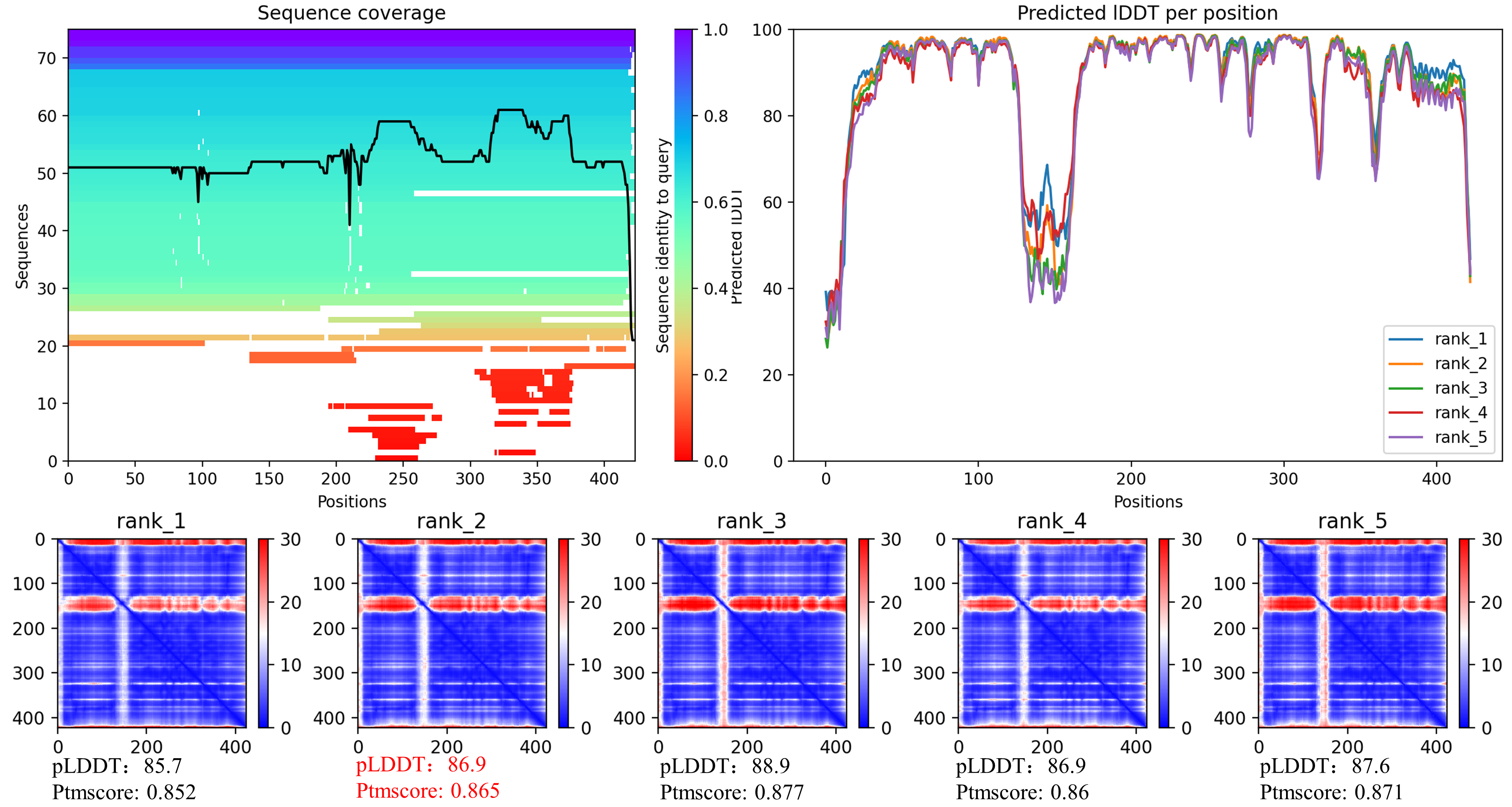

Supplement: S11 Fig — (TIF) [file pone.0303501.s013.tif]

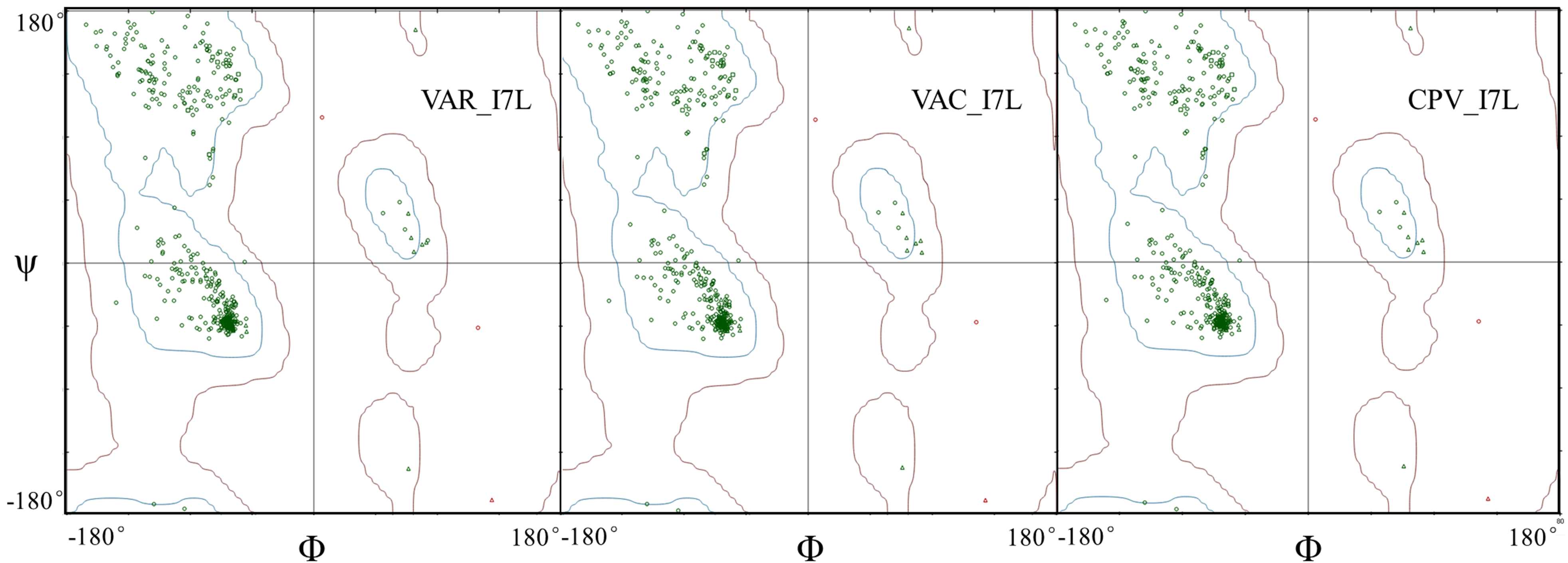

Supplement: S12 Fig — (TIF) [file pone.0303501.s014.tif]

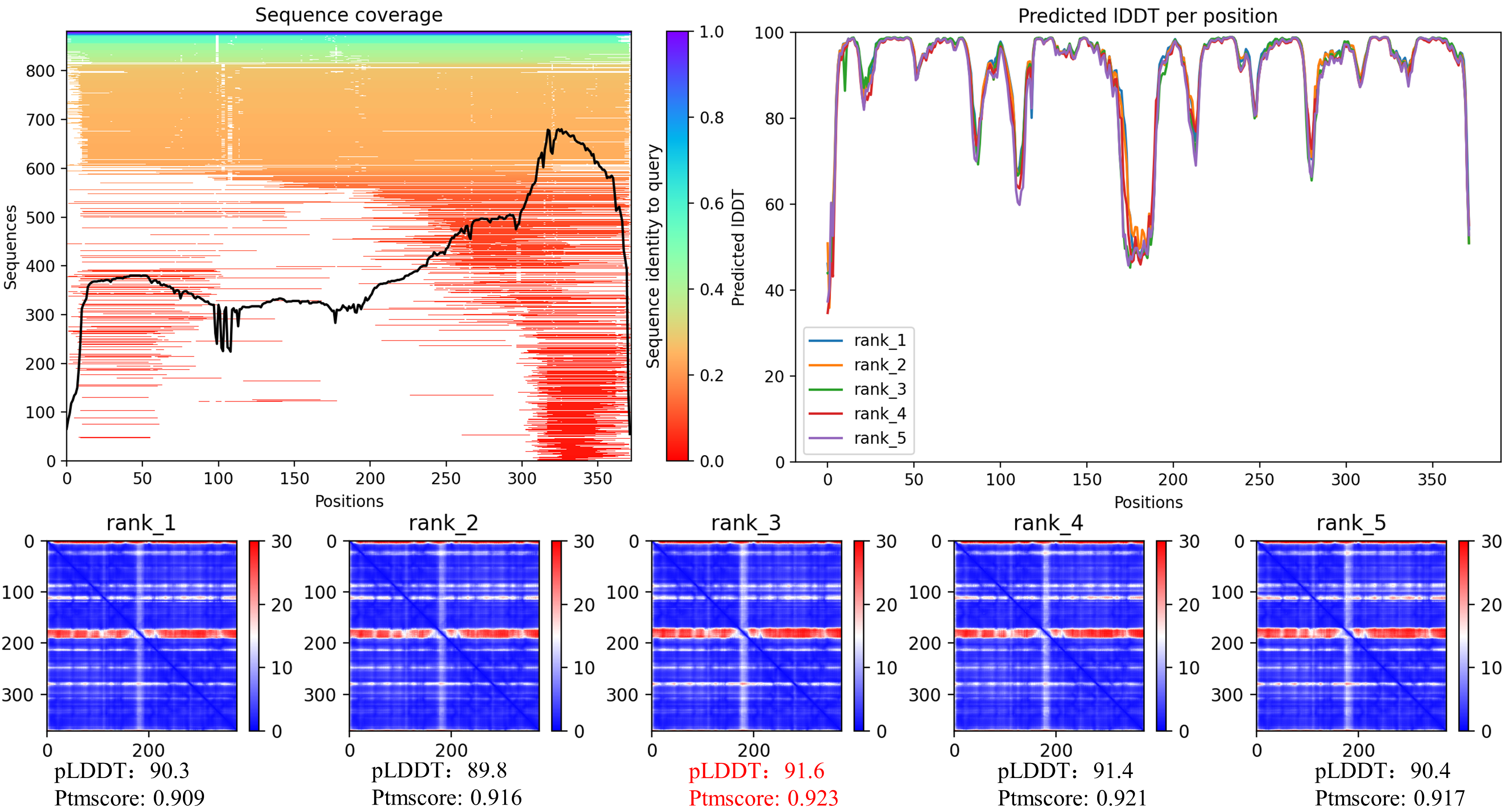

Supplement: S13 Fig — (TIF) [file pone.0303501.s015.tif]

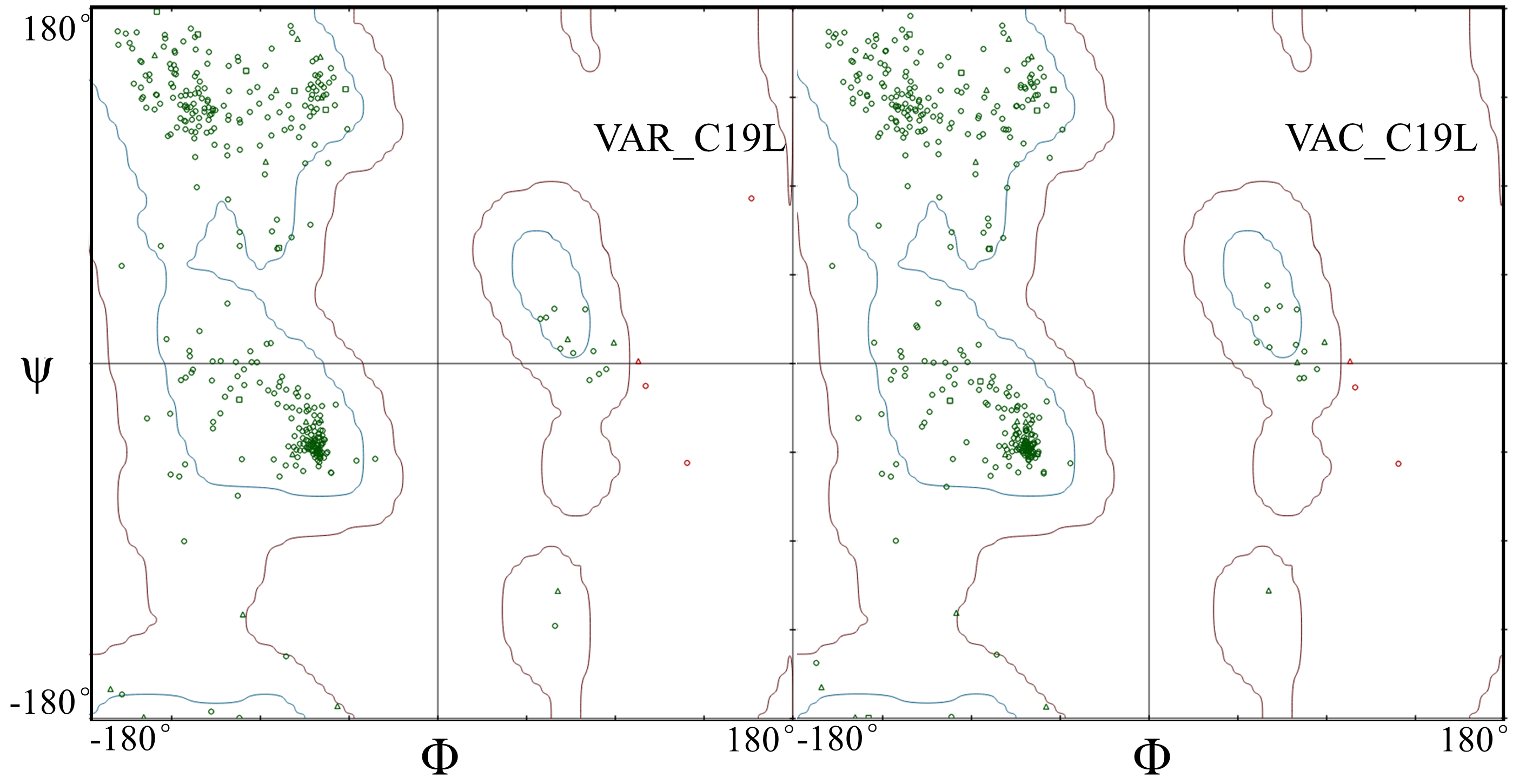

Supplement: S14 Fig — (TIF) [file pone.0303501.s016.tif]

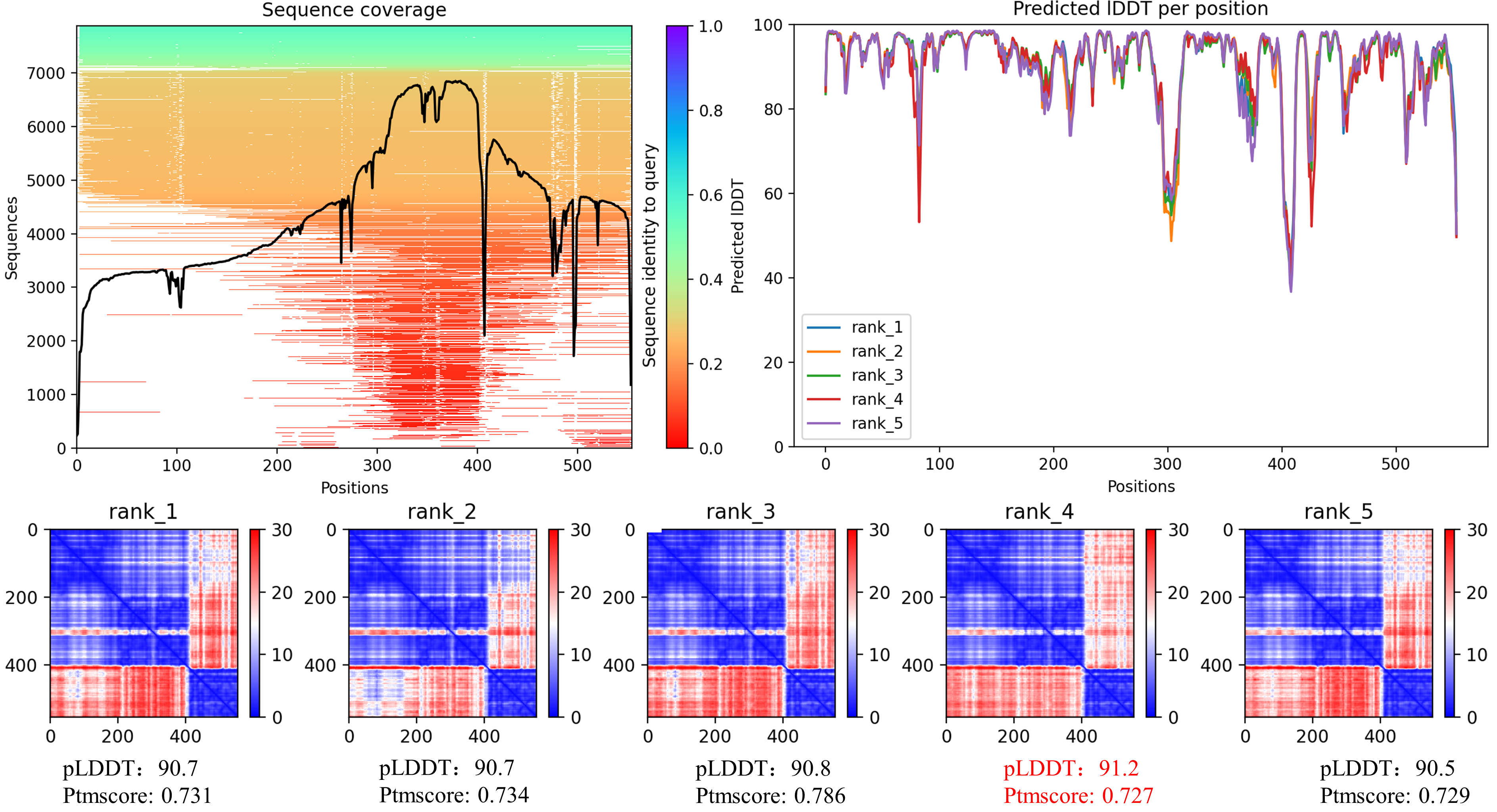

Supplement: S15 Fig — (TIF) [file pone.0303501.s017.tif]

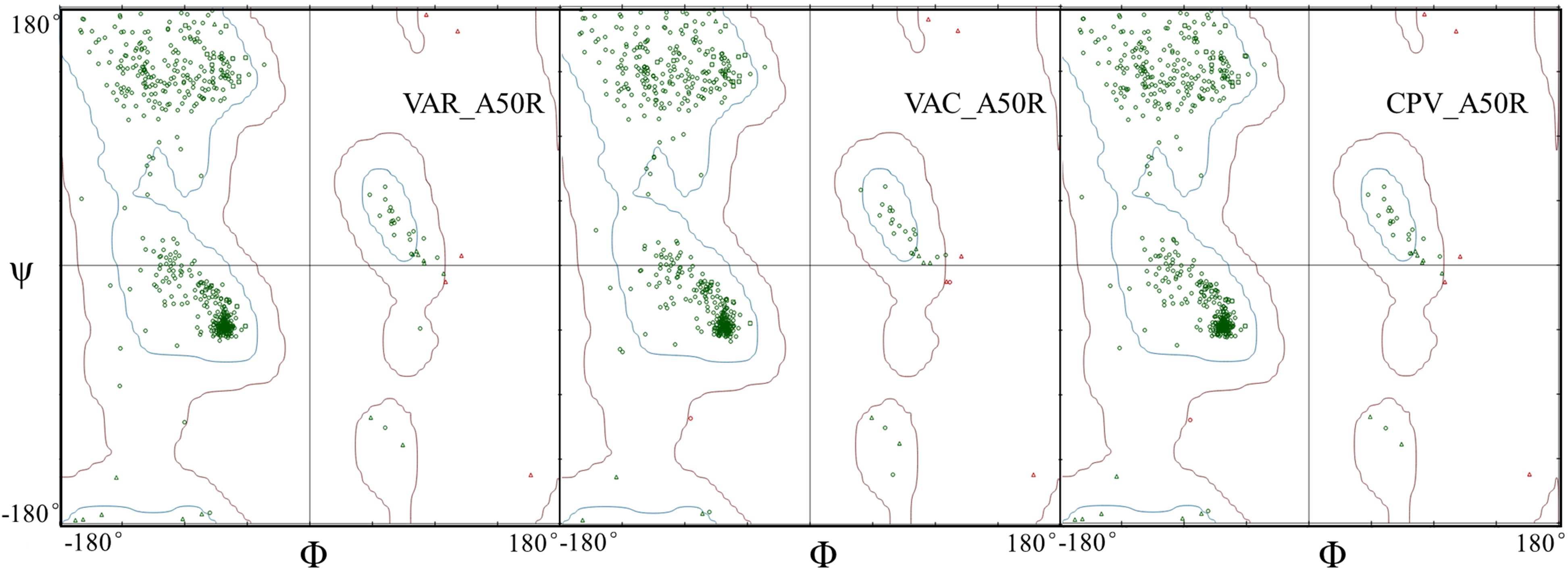

Supplement: S16 Fig — (TIF) [file pone.0303501.s018.tif]
