# Supplementary material for: Identification of core therapeutic targets for Monkeypox virus and repurposing potential of drugs: A WEB prediction approach
Source: PLoS One. 2024 Dec 6;19(12):e0303501. doi: 10.1371/journal.pone.0303501 (PMC11623562; doi:10.1371/journal.pone.0303501)
Supplement: Attachment — Submitted filename: Comments_ PONE_D_24_03917.doc [file pone.0303501.s019.doc]

**Development of mechanism‐ based inhibitor against Monkeypox virus: A WEB to predict the binding patterns**

**Reviewer’s comments and recommendations:**

In this study the authors claim to have developed a monkeypox core protein library and a library of inhibitors targeting these core proteins. The authors have in addition built a docking server that predicts the binding modes between the protein and the substrate to understand the molecular dialogues between these entities. Currently there are hardly such servers dedicated for monkeypox virus. Therefore, such an endeavour is greatly welcome. Although the paper appears to be sound overall, I have some key recommendations that that I think can be incorporated before the paper can be accepted for publication.

1. In the methodology section method 2.1.Open reading frame (ORF), the ORF needs to be defined more concretely. I advise the authors to have a look at the results section (3.1 The meaning of the open reading frame (ORF) and core proteins in this study) of the paper titled “Identification of core therapeutic targets for Monkeypox virus andrepurposing potential of drugs against them: An in silico approach”for better clarity.
2. Similarly, the term core protein creates a lot of confusion. From the view of a genomics researcher core protein is more closely associated with proteins that are highly conserved across all the genomes. However, from the point of view of a classical virologist core protein usually means the structural protein embedded on the outer membranes that give a virus its definitive shape. Therefore, the authors should clearly mention this in the manuscript as readers will comprise of people from multiple disciplines. Again, I advise the authors to have a look at the results section (3.1 The meaning of the open reading frame (ORF) and core proteins in this study) of the paper titled “Identification of core therapeutic targets for Monkeypox virus and repurposing potential of drugs against them: An in silico approach”for better clarity.
3. Shared host homology is one of the biggest reasons for the failure of a drug from reaching the bed from the bench. This often creates adverse reactions by to off-targeting the host proteins instead. As a consequence, drugs often fail during clinical trials. Therefore, the authors must verify that the 12 key proteins do not share homology with human proteins beyond an allowable threshold (usually e-value of 0.0001 against the entire set of human proteome dataset (taxid: 9606) is considered a good threshold). The authors must provide this information in the manuscript.
4. Why only 12 proteins were selected as targets? Are all the 12 key proteins druggable? The authors need to verify this information as not all proteins present highly druggable surfaces for docking. The validity of the structures used for docking should be more adequately demonstrated.
5. Are any of the proteins intrinsically disordered? This is a very key question that needs answering. They are a fascinating class of proteins that lack a fixed or well-defined three-dimensional structure in their free state. Therefore, docking against such proteins are more challenging than what meets the eye. Therefore, physiochemical properties like isoelectric point (pI), molecular weight, aliphatic index, instability index, extinction co-efficient and residues accessibility of the 12 key proteins must be examined and provided in the manuscript. This data will aid experimental biologists to choose suitable proteins for experimental validation.

Overall, the proteins that are highly conserved, non-host homologous, highly druggable are usually	considered very high value therapeutic targets. The proteins that ensure these features are likely to	have a higher rate of success. Hence, I feel these key points need to be investigated by the authors	before being considered for final consideration. The above recommended paper should aid the	authors in this endeavour.

6. The efficiency of the docking server is not demonstrated with appropriate controls. Since, none of the results are experimentally validated, it is necessary to demonstrate the efficiency by comparison with other docking models.
