# Supplementary material for: Identification of core therapeutic targets for Monkeypox virus and repurposing potential of drugs: A WEB prediction approach
Source: PLoS One. 2024 Dec 6;19(12):e0303501. doi: 10.1371/journal.pone.0303501 (PMC11623562; doi:10.1371/journal.pone.0303501)
Supplement: Attachment — Submitted filename: Response to reviewers.docx [file pone.0303501.s020.docx]

Prof. Jianping Hu, PhD

Chengdu University

610106 Chengdu, China

E-mail: hjpcdu@163.com

Chengdu, January 18^th^, 2024

Dear Editorial Office

Editor, ***PLOS ONE***

Thank you very much for your email of Major decision, regarding our manuscript ***Development of mechanism‐based inhibitor against Monkeypox virus: A WEB to predict the binding patterns* (Manuscript Number: PONE-D-24-03917R1).**

We would like to thank wholeheartedly the referees for their valuable and helpful comments about our manuscript. Thanks to the reviewers for their recognition of the research objectives, rationale and methods of the article, as well as confirming the experimental data of the article. Suggestions on the figures and structure of the article will also be listened to and improved upon. Our replies to reviewers’ comments are enclosed below and in the revised version of the manuscript, we marked two parts in red: the suggestions given by the reviewers and the contents modified by the second reading of the article. We hope that our revised manuscript is now suitable for publication in ***PLOS ONE.***

Yours sincerely,

Dr. Jianping Hu

E-mail: hjpcdu@163.com

**Reply to Editor**

Thank you to the editors for their advice on article acceptance. We have reconfirmed PLOS-ONE's requirements for articles as well as shared code to ensure that the submitted manuscripts are in order. Second, the funder had no role in the study design, data collection and analysis, publication decision, or preparation of the manuscript. Additionally, the editor mentioned a data sharing program, and we accept the editor's suggestion that we can accept sharing of data prior to article acceptance. The editor also raised the issue of copyright, and we were acutely aware of the importance of copyright, so we corrected the article and removed Figure s.17 Finally, for the supporting material we made a separate file and made sure that each information file had a legend in the manuscript following the list of references.

**Reply to Reviewer 1**

In this study the authors claim to have developed a monkeypox core protein library and a library of inhibitors targeting these core proteins. The authors have in addition built a docking server that predicts the binding modes between the protein and the substrate to understand the molecular dialogues between these entities. Currently there are hardly such servers dedicated for monkeypox virus. Therefore, such an endeavor is greatly welcome. Although the paper appears to be sound overall, I have some key recommendations that that I think can be incorporated before the paper can be accepted for publication.

**Specific comment** 1

In the methodology section method 2.1. Open reading frame (ORF), the ORF needs to be defined more concretely. I advise the authors to have a look at the results section (3.1 The meaning of the open reading frame (ORF) and core proteins in this study) of the paper titled &ldquo; Identification of core therapeutic targets for Monkeypox virus and repurposing potential of drugs against them: An in silico approach &rdquo;for better clarity.

**Response** 1

I appreciate the editor's comments and I have taken the time to check out the full article. The Open Reading Frame (ORF) serves as the beginning of the article and has an important role in the subsequent work. In the Methods section we considered the length of the article, so we simply explained the ORF. In response to the editor's suggestion, we have explained this section in more detail, and the specific corrections are as follows:

ORF is a fundamental concept in molecular bioinformatics, it is the normal nucleotide sequence of a structural gene, the reading frame from the start codon to the stop codon can encode a complete polypeptide chain, between which there is no stop codon that interrupts the translation. The term open refers to the "open" region of an intact gene used for protein translation, and reading frame refers to one of the six possibilities for translation of a double-stranded gene sequence into amino acids. In molecular biology, the detection of ORFs is an important step in the discovery of specific protein-coding genes in the genome sequence, and can serve as an indicator of a potential protein-coding gene. In our work, the acquisition of ORF mainly includes literature reading and database research.

In addition to the suggestions for ORFs, the editors also made a suggestion to change the title of the article to "Identification of core therapeutic targets for Monkeypox virus and repurposing potential of drugs against They: An in-silico approach", which is more in accordance with the content of the article. In our very first title, we targeted the server, ignoring the fact that the article studies more on monkeypox virus and core proteins. Seeing the editor's suggestion, we appreciate being able to identify this issue and also think that changing the title would benefit the article more.

**Specific comment** 2

Similarly, the term core protein creates a lot of confusion. From the view of a genomics researcher core protein is more closely associated with proteins that are highly conserved across all the genomes. However, from the point of view of a classical virologist core protein usually means the structural protein embedded on the outer membranes that give a virus its definitive shape. Therefore, the authors should clearly mention this in the manuscript as readers will comprise of people from multiple disciplines. Again, I advise the authors to have a look at the results section (3.1 The meaning of the open reading frame (ORF) and core proteins in this study) of the paper titled “Identification of core therapeutic targets for Monkeypox virus and repurposing potential of drugs against them: An in-silico approach” for better clarity.

**Response** 2

We thank the reviewers for this suggestion, and we do recognize the reviewers' suggestion that the core proteins were improperly worded. In our work, core proteins are used to denote potential proteins with the potential to inhibit monkeypox virus, and do not refer to structural proteins or highly conserved proteins as mentioned by the reviewer. In order to avoid misunderstanding, we have corrected all references to core proteins in the article and replaced the original core proteins with potential targets. The following are some of the corrections:

A new round of monkeypox virus has emerged in the United Kingdom since July 2022 and rapidly swept the world. Currently, despite numerous research groups are studying this virus and seeking effective treatments, the information on the open reading frame, inhibitors, and potential targets of monkeypox has not been updated in time, and the comprehension of monkeypox target ligand interactions remains a key challenge. Here, we first summarized and improved the open reading frame information of monkeypox, constructed the monkeypox inhibitor library and potential targets library by database research as well as literature search, combined with advanced protein modeling technologies (Sequence-based and AI algorithms-based homology modeling). In addition, we build monkeypox virus Docking Server, a web server to predict the binding mode between targets and substrate. The open reading frame information, monkeypox inhibitor library, and monkeypox potential targets library are used as the initial files for server docking, providing free interactive tools for predicting ligand interactions of monkeypox targets, potential drug screening, and potential targets search. In addition, the update of the three databases can also effectively promote the study of monkeypox drug inhibition mechanism and provide theoretical guidance for the development of drugs for monkeypox.

In this issue, the reviewer again proposed a change in the title of the article. In the previous comment, we clearly stated that we recognized the reviewer's title and explained the reason for the title in the first place. Again, we thank the reviewers for their attention to this issue.

**Specific comment** 3

Shared host homology is one of the biggest reasons for the failure of a drug from reaching the bed from the bench. This often creates adverse reactions by to off-targeting the host proteins instead. As a consequence, drugs often fail during clinical trials. Therefore, the authors must verify that the 12 key proteins do not share homology with human proteins beyond an allowable threshold (usually e-value of 0.0001 against the entire set of human proteome dataset (taxid: 9606) is considered a good threshold). The authors must provide this information in the manuscript.

**Response** 3

This question raised by the reviewer is very interesting and we have addressed it with a detailed explanation. Shared host homology as an important issue that must be considered for subsequent drug development, if the homology is too high it will lead to poor drug selectivity, and attacking the viral cells while also harming the host cells to a certain extent. In our article, this issue was neglected, so in the revised manuscript, for both host and viral cells we added this part. This includes calculation of physicochemical information of 12 proteins, sequence similarity with human-derived proteins, VAC, VAR and CPV, detailing the host homology issue.

The three-dimensional structure of proteins is an important basis for understanding their biological functions and for structure-based drug design [104]. With the rapid development of structural biology, the speed of measuring protein 3D structures using methods such as NMR, X-Ray or cryoelectronic microscopy (Cryo-EM) crystallography has been greatly improved [39, 105]. However, the 3D structures of some proteins are still difficult to determine due to many reasons, such as excessive molecular weight or difficulty in crystallization. Before modeling the protein structures, we first performed a series of physicochemical property analyses on 12 potential target sequences, including Weight, PI, Extinction, Instability, Aliphatic and Hydrophathicity, and compared the sequences of monkeypox with those of human origin and other orthopox viruses (table s1). It was found that the structural domains of the proteins were stabilized to a certain extent and could be used for subsequent studies; The sequence similarity of all proteins except D6L was less than 60% to human, which avoided the side effects of the drug caused by host homology. D6L, as an interleukin-18 binding protein homologous to the host, functionally affirmed its high sequence similarity to human proteins, a characteristic that also increased the difficulty of developing drugs against it; finally, the sequence similarity between monkeypox virus and orthopoxvirus was higher than 78%, proving the existence of commonality between them, and providing an opportunity to seek possible orthopoxvirus-based drugs for the subsequent studies. This lays a theoretical foundation for the search of possible monkeypox virus drugs based on orthopox viruses.

For the key proteins of monkeypox, we found that eight (A49R, C3L, H1L, E4R, C7L, A41L, D6L and E13L) could be obtained from orthopox viruses (VAR, VAC and MPV) modeled with high sequence similarity. As for the key proteins (A22R, I7L, C19L and A50R), which are not present in any of the orthopoxvirus genera, we chose AlphaFold2, which is only one atomic width away from the true structure for most of the predicted protein structures, to predict them. Notably that based on the multifaceted superiority, AlphaFold2 reaches the level of prediction observed by sophisticated instruments such as cryoelectron microscopy, which can effectively guarantee the accuracy of the predicted structures (Fig.2) [106]. Hence, we constructed a database of orthopoxvirus proteins with a capacity of 45.

Table s1 Physicochemical properties of 12 potential target protein sequences and sequence similarity to human and orthopoxvirus

|  | A49R | H1L | C7L | D6L | A22R | C19L | C3L | E4R | A41L | E13L | I7L | A50R |
| --- | --- | --- | --- | --- | --- | --- | --- | --- | --- | --- | --- | --- |
| Weight | 23289.7 | 19727.8 | 25705.3 | 14337.6 | 49147.1 | 41808.7 | 4982.7 | 25076.8 | 25371.9 | 61937.0 | 79605.2 | 63574.3 |
| PI | 5.3 | 9.2 | 4.7 | 6.5 | 5.6 | 6.5 | 4.3 | 7.0 | 5.0 | 5.2 | 7.5 | 7.5 |
| Extinction | 28420 | 17880 | 28310 | 18450 | 49280 | 52370 | 8940 | 45380 | 24870 | 58680 | 72660 | 58790 |
| Instability | 50.4 | 46.8 | 41.4 | 29.0 | 36.3 | 29.8 | 29.7 | 42.1 | 39.4 | 28.8 | 30.0 | 32.2 |
| Aliphatic | 90.8 | 86.0 | 84.1 | 82.7 | 92.8 | 93.8 | 95.3 | 92.1 | 81.9 | 90.1 | 86.4 | 90.0 |
| Hydrophathicity | -0.245 | -0.308 | -0.248 | -0.247 | -0.258 | -0.112 | 0.160 | -0.258 | -0.360 | -0.200 | -0.236 | -0.344 |
| Human Identity | 42.8 | 26.9 | <10% | 94.3 | <10% | 16.6 | 24.2 | <10% | <10% | 26.7 | 19.6 | 56.6 |
| VAR Identity | 98.5 | 98.8 | 83.6 | 92.1 | 96.7 | 97.3 | 78.6 | 96.8 | 91.4 | 98.9 | 98.8 | 96.4 |
| VAC Identity | 99.0 | 100.0 | 83.6 | \ | 97.4 | 98.7 | 95.2 | 98.6 | 94.1 | 98.9 | 99.1 | 97.8 |
| CPV Identity | 99.0 | 98.8 | 87.2 | 79.7 | \ | 98.7 | 97.6 | \ | 94.1 | \ | \ | 97.8 |

**Specific comment** 4

Why only 12 proteins were selected as targets? Are all the 12 key proteins druggable? The authors need to verify this information as not all proteins present highly druggable surfaces for docking. The validity of the structures used for docking should be more adequately demonstrated.

**Response** 4

We are grateful to the reviewers for raising the issue of proteins druggable, and we will provide answers to each of the questions raised. Firstly, about the 12 target proteins selected: in the preliminary background research, this work extensively read all the articles (more than 1,000) on orthopox viruses since the first outbreak to seek for key targets that might inhibit orthopox viruses. During infection, a large number of proteins are involved in the process of virus invasion, replication, envelopment and release, and the virus can be effectively inhibited by inhibiting these key proteins. Based on literature research, 12 key proteins with important roles for the virus were finally searched and identified by summarizing the studies of various infection processes. The whole work does not involve screening proteins, but rather investigating all the targets that have been clearly shown to be potential in studies. The second problem is for the proteins druggable: the treatment of monkeypox is still in the exploratory stage, and there is no specific drug that can effectively deal with it. The 12 target proteins are all shown to be potentially druggable, and each protein is clearly described in section 3.2 of the article, explaining why it is a potential target for monkeypox treatment. In addition, all 12 proteins have clear docking sites, which were identified by previous researchers through various chemical experiments, not just theoretical descriptions. So, in response to the reviewer's question about the validity of the docking structure I think there is no problem.

**Specific comment** 5

Are any of the proteins intrinsically disordered? This is a very key question that needs answering. They are a fascinating class of proteins that lack a fixed or well-defined three-dimensional structure in their free state. Therefore, docking against such proteins are more challenging than what meets the eye. Therefore, physiochemical properties like isoelectric point (pI), molecular weight, aliphatic index, instability index, extinction co-efficient and residues accessibility of the 12 key proteins must be examined and provided in the manuscript. This data will aid experimental biologists to choose suitable proteins for experimental validation.

Overall, the proteins that are highly conserved, non-host homologous, highly druggable are usually considered very high value therapeutic targets. The proteins that ensure these features are likely to have a higher rate of success. Hence, I feel these key points need to be investigated by the authors before being considered for final consideration. The above recommended paper should aid the authors in this endeavor.

**Response** 5

We recognize in depth the issues raised by the reviewers. Among the 12 proteins we studied, there is no disordered protein structure. The three-dimensional structure of proteins serves as an important basis for understanding their biological functions and structure-based drug design. Using homologous mode-bonding technique, we obtained monkeypox target protein structures based on eight (A49R, C3L, H1L, E4R, C7L, A41L, D6L, and E13L) orthopoxviral (VAR, VAC, and CPV) proteins that were already available as templates. As for the key proteins (A22R, I7L, C19L and A50R), which are not present in any of the orthopoxvirus genera, we chose AlphaFold2, which is only one atomic width away from the true structure for most of the predicted protein structures, to predict them. Notably that based on the multifaceted superiority, AlphaFold2 reaches the level of prediction observed by sophisticated instruments such as cryoelectron microscopy, which can effectively guarantee the accuracy of the predicted structures

In addition to the acquisition of protein structures, the reviewers suggested to supplement the information on the physical and chemical parameters of the target proteins. We were happy to accept the reviewer's comments and immediately analyzed the 12 proteins for their physical chemistry information. As shown in Table 2, we demonstrated six physical parameters of the 12 proteins, including Weight, PI, Extinction, Instability, Aliphatic and Hydrophathicity, and each parameter was in a reasonable range, indicating that the structural domains of the proteins were stabilized to a certain extent and could be used for subsequent studies. In addition, the reviewers again mentioned highly conserved, non-host homologous and highly druggable, which we have analyzed in detail in question 3 and demonstrated these properties of the system. Finally, we would like to thank the reviewers again for their pertinent suggestions, which have been corrected in the article. Followed by a corrected version in the article

The three-dimensional structure of proteins is an important basis for understanding their biological functions and for structure-based drug design [104]. With the rapid development of structural biology, the speed of measuring protein 3D structures using methods such as NMR, X-Ray or cryoelectron microscopy (Cryo-EM) crystallography has been greatly improved [39, 105]. However, the 3D structures of some proteins are still difficult to determine due to many reasons, such as excessive molecular weight or difficulty in crystallization. Before modeling the protein structures, we first performed a series of physicochemical property analyses on 12 potential target sequences, including Weight, PI, Extinction, Instability, Aliphatic and Hydrophathicity, and compared the sequences of monkeypox with those of human origin and other orthopox viruses (table s1). It was found that the structural domains of the proteins were stabilized to a certain extent and could be used for subsequent studies; The sequence similarity of all proteins except D6L was less than 60% to human, which avoided the side effects of the drug caused by host homology. D6L, as an interleukin-18 binding protein homologous to the host, functionally affirmed its high sequence similarity to human proteins, a characteristic that also increased the difficulty of developing drugs against it; finally, the sequence similarity between monkeypox virus and orthopoxvirus was higher than 78%, proving the existence of commonality between them, and providing an opportunity to seek possible orthopoxvirus-based drugs for the subsequent studies. This lays a theoretical foundation for the search of possible monkeypox virus drugs based on orthopox viruses.

For the key proteins of monkeypox, we found that eight (A49R, C3L, H1L, E4R, C7L, A41L, D6L and E13L) could be obtained from orthopox viruses (VAR, VAC and MPV) modeled with high sequence similarity. As for the key proteins (A22R, I7L, C19L and A50R), which are not present in any of the orthopoxvirus genera, we chose AlphaFold2, which is only one atomic width away from the true structure for most of the predicted protein structures, to predict them. Notably that based on the multifaceted superiority, AlphaFold2 reaches the level of prediction observed by sophisticated instruments such as cryoelectron microscopy, which can effectively guarantee the accuracy of the predicted structures (Fig.2) [106]. Hence, we constructed a database of orthopoxvirus proteins with a capacity of 45.

Table s1 Physicochemical properties of 12 potential target protein sequences and sequence similarity to human and orthopoxvirus

|  | A49R | H1L | C7L | D6L | A22R | C19L | C3L | E4R | A41L | E13L | I7L | A50R |
| --- | --- | --- | --- | --- | --- | --- | --- | --- | --- | --- | --- | --- |
| Weight | 23289.7 | 19727.8 | 25705.3 | 14337.6 | 49147.1 | 41808.7 | 4982.7 | 25076.8 | 25371.9 | 61937.0 | 79605.2 | 63574.3 |
| PI | 5.3 | 9.2 | 4.7 | 6.5 | 5.6 | 6.5 | 4.3 | 7.0 | 5.0 | 5.2 | 7.5 | 7.5 |
| Extinction | 28420 | 17880 | 28310 | 18450 | 49280 | 52370 | 8940 | 45380 | 24870 | 58680 | 72660 | 58790 |
| Instability | 50.4 | 46.8 | 41.4 | 29.0 | 36.3 | 29.8 | 29.7 | 42.1 | 39.4 | 28.8 | 30.0 | 32.2 |
| Aliphatic | 90.8 | 86.0 | 84.1 | 82.7 | 92.8 | 93.8 | 95.3 | 92.1 | 81.9 | 90.1 | 86.4 | 90.0 |
| Hydrophathicity | -0.245 | -0.308 | -0.248 | -0.247 | -0.258 | -0.112 | 0.160 | -0.258 | -0.360 | -0.200 | -0.236 | -0.344 |
| Human Identity | 42.8 | 26.9 | <10% | 94.3 | <10% | 16.6 | 24.2 | <10% | <10% | 26.7 | 19.6 | 56.6 |
| VAR Identity | 98.5 | 98.8 | 83.6 | 92.1 | 96.7 | 97.3 | 78.6 | 96.8 | 91.4 | 98.9 | 98.8 | 96.4 |
| VAC Identity | 99.0 | 100.0 | 83.6 | \ | 97.4 | 98.7 | 95.2 | 98.6 | 94.1 | 98.9 | 99.1 | 97.8 |
| CPV Identity | 99.0 | 98.8 | 87.2 | 79.7 | \ | 98.7 | 97.6 | \ | 94.1 | \ | \ | 97.8 |

**Specific comment** 6.

The efficiency of the docking server is not demonstrated with appropriate controls. Since, none of the results are experimentally validated, it is necessary to demonstrate the efficiency by comparison with other docking models.

**Response** 6

We thank the reviewers for their suggestions on the manuscript, which we strongly acknowledge: the rate issue for the server is indeed what we lacked. The docking sever is based on CentOS for Linux, the website application is deployed using Docker The docking sever is based on CentOS for Linux, the website application is deployed using Docker containerization technology, and the website requests on port 80 are proxied and forwarded through NGINX, which ensures that users get the docking result quickly after submitting the docking task. In our example, we also completed the docking task for the small molecule inhibitor database in just over 20 minutes. In addition, the reviewer suggested that the article did not have results that were experimentally validated. I think there might be a misunderstanding of our database by the reviewer, all the small molecules are potential inhibitors based on experimental data, take C13L protein as an example: with the prevalence of monkeypox in recent years, Yuen found that ST246 can effectively inhibit orthopox viruses, including monkeypox virus, and inhibit a variety of mutations. In addition, it is safe and well tolerated with no serious side effects. The library of inhibitors collected in the manuscript are compounds that have been experimentally validated for their potential inhibitory activity. For model validation, we utilized a reverse molecular docking approach, where all small molecule inhibitors were simultaneously docked to 12 possible targets to verify the accuracy of their active sites. The following are corrections to the article:

Table 2 gives the methods for obtaining the 12 key protein structures, the types of modeled protein structures and the scoring results of the AplhaFold2 modeled structures. It can be found that basically all the orthopox virus genus proteins can be reasonably molded and the simulated structures are relatively reasonable. Figures s1-s16 show the reasonableness of the modeled structures of each system. Taking the A22R modeling results as an example (Fig. s9, s10), in the AlphaFold2 modeling results, rank_4 has the highest pLDDT and Ptmscore for the five modeled structures given, which means that this structure is the most representative of the dominant conformation of A22R. Specifically, from the Sequence coverage and predicted IDDT, it is easy to find that the residues of the protein are in high Sequence identity and more than 98 residues are in high pIDDT values (pIDDT > 60) [107]. Based on rank_4, we used Discovery Studio to model the VAR, VAC, and MPV, and the results showed that over 95% of the amino acids were in the conformational optimum region, and only a few amino acids (VAR:5, VAC:7, MPV:6) were in the conformational inappropriate region. Considering that the protein contains more than 400 amino acids and the amino acids in the unreasonable region are in the loop region, which is far from the core region of the protein, it is considered reasonable to build the conformation [28, 108]. In addition, further reverse molecular docking work was used to verify the rationality of the protein structure. Through the Autodock vina program package, we docked 189 small molecules to each protein separately. As shown in Table 2, more than 135 (more than 68%) were docked with affinity less than -5 for each system, indicating that the protein active site is relatively reasonable and can ensure the follow-up work.

Table 2 Structure acquisition methods, structural soundness scoring and docking success rate of 12 key proteins

| ORFs | Methods | Protein Category | pLDDT | Rate%* | ORFs | Methods | Protein Category | pLDDT | Rate% |
| --- | --- | --- | --- | --- | --- | --- | --- | --- | --- |
| A49R | RCSB, DS | **VAR**, VAC, CPV, MPV | - | 78.79 | C3L | RCSB, DS | VAR, **VAC**, CPV | - | 82.32 |
| H1L | RCSB, DS | VAR, **VAC**, CPV, MPV | - | 68.19 | E4R | RCSB, DS | VAR, **VAC**, CPV, MPV | - | 77.78 |
| C7L | RCSB, DS | **VAR**, VAC, CPV, MPV | - | 83.33 | A41L | RCSB, DS | **VAR**, VAC, CPV, MPV | - | 73.23 |
| D6L | RCSB, DS | VAR, VAC, CPV, MPV | - | 79.30 | E13L | RCSB, DS | VAR, **VAC**, CPV, MPV | - | 76.26 |
| A22R | AlphaFold2, DS | VAR, VAC, **MPV** | 90.3 | 85.29 | I7L | AlphaFold2, DS | VAR, VAC, CPV, **MPV** | 86.9 | 82.32 |
| C19L | AlphaFold2, DS | VAR, VAC, **MPV** | 91.6 | 75.25 | A50R | AlphaFold2, DS | VAR, VAC, CPV, **MPV** | 91.2 | 69.70 |

*: Proportion of docking results with docking affinity below -0.5 in the small molecule database

**Reply to Reviewer 2**

The paper titled “Development of mechanism‐based inhibitor against Monkeypox virus: A WEB to predict the binding patterns” is like a review on the said “Monkeypox virus Docking Server”. The server might serve as a good resource for people working on the virus, which is indeed comprehensive and well-thought-out. It can perform modeling, docking, and annotation. Still, it lacks a firm basis, and improvement is needed. The issues pointed out below are critical and need to be properly addressed

**Specific comment** 1

The key to publishing a paper on a developed tool is to put forth a detailed analysis concerning the accuracy of the results yielded by it through statistical inferences. The authors have presented an example but it's not enough to validate the potentiality of the server.

**Response** 1

We are grateful to the reviewers for their professional comments, and we have received the questions raised by the reviewers with an open mind. In our actual work, we predicted all 12 systems, and the results indicated that the substrates were able to bind to the proteins well (the docking energies of the top 10 Ranks were all less than -6.00), and the binding sites were consistent with the literature. Considering the length of the article and the fact that all the articles on the same type of website showed only one example, we randomly chose a set of results to show in the final writing. In addition to the server work, we are also synchronizing our in-depth research on C13L and ST246.

**Specific comment** 2

It is clear that the server provides functional annotations for specific genes or proteins of interest, but are there tools or functionalities available for analyzing gene function, protein structure, or pathway enrichment? How does the server facilitate the comparative analysis of genes, proteins, or genomes across different ortho-poxviruses? It would be interesting if there were such a provision that the server could carry out.

**Response** 2

Thanks to the reviewers for their suggestions. In our server, the screening work for targets or potential small molecule drugs is mainly realized by molecular docking. For now, tools for gene function, protein structure, pathway enrichment, etc. are not involved. Currently, there are a large number of platforms for these tools that have been widely promoted and have proved the reasonableness of the results in the process of continuous use, including: KEGG, NCBI, RCSB and so on. As a drug screening platform, we hope to focus on docking work, and we also accept the reviewers' suggestions, and will open a new partition - commonly used tools partition later. Under this partition, profiles and links to some commonly used tools for gene function, protein structure, and pathway enrichment will be provided. Similarly, the reviewer mentioned the comparative analysis of genes, proteins or genomes of different orthopox viruses, and we will also add a brief introduction and links to common tools for gene comparison, protein comparison and genome comparison in the section of Common Tools.

**Specific comment** 3

Have the authors conducted site-specific docking or blind docking methodologies in this investigation? Please provide a clear explanation. It's not clear how, in case of no active site details, the tool can locate an active site for a newly modeled protein by reverse docking. The authors need to elaborate on that.

**Response** 3

For the docking work, we thank the reviewers for their professional advice. Molecular docking, as a process of simulated molecular recognition in computers, can quickly screen the optimal binding conformations of proteins and their ligands, and the identified active site information can effectively ensure the rationality of the results. In our work, through extensive literature research, we provided the key amino acid composition of the active centers for all protein structures (Table 1), and the accurate binding pockets ensure the effectiveness of the subsequent drug screening work. In addition, for all protein structures, we realized reverse molecular docking through several molecular docking software (DS, FlexX, Autodock), which verified the active site and also ensured the reasonableness of the modeled protein structures. In this manuscript, this part was ignored considering the article length article. We have decided to add this section to the main text through the suggestions of the reviewers, and the specific corrections are as follows:

Table 2 gives the methods for obtaining the 12 key protein structures, the types of modeled protein structures and the scoring results of the AplhaFold2 modeled structures. It can be found that basically all the orthopox virus genus proteins can be reasonably molded and the simulated structures are relatively reasonable. Figures s1-s16 show the reasonableness of the modeled structures of each system. Taking the A22R modeling results as an example (Fig. s9, s10), in the AlphaFold2 modeling results, rank_4 has the highest pLDDT and Ptmscore for the five modeled structures given, which means that this structure is the most representative of the dominant conformation of A22R. Specifically, from the Sequence coverage and predicted IDDT, it is easy to find that the residues of the protein are in high Sequence identity and more than 98 residues are in high pIDDT values (pIDDT > 60) [107]. Based on rank_4, we used Discovery Studio to model the VAR, VAC, and MPV, and the results showed that over 95% of the amino acids were in the conformational optimum region, and only a few amino acids (VAR:5, VAC:7, MPV:6) were in the conformational inappropriate region. Considering that the protein contains more than 400 amino acids and the amino acids in the unreasonable region are in the loop region, which is far from the core region of the protein, it is considered reasonable to build the conformation [28, 108]. In addition, further reverse molecular docking work was used to verify the rationality of the protein structure. Through the Autodock vina program package, we docked 189 small molecules to each protein separately. As shown in Table 2, more than 135 (more than 68%) were docked with affinity less than -5 for each system, indicating that the protein active site is relatively reasonable and can ensure the follow-up work.

Table 1 Isologs, function and residue information of 12 key proteins in MPV replication

| ORFs | Isologs | Function | Key residues |
| --- | --- | --- | --- |
| A49R | VAR/J2L, VAC/A48R, CPV/A51R | Thymidine kinase | R41, D13, Y144 |
| C3L | VAR/C3L, VAC/K3L, CPV/M3L | Eukaryotic translation initiation factor-2a homolog protein | A25, Y27, I39 |
| H1L | VAR/I1L, VAC/H1L | Tyrosine/serine protein phosphatase, blocks IFN-γ signal | N115, G113, V114 |
| E4R | VAR/F4R, VAC/D4R | Uracil DNA glycosylase | G215, F216,Y218 |
| C7L | VAR/C5L, VAC/F1L, CPV/G1L | Hypothrtical protein | V115, L158, G155 |
| A41L | VAR/A46L, VAC/A41L, CPV/A43L | Secreted protein reduces influx of inflammatory cells | F21, L47, F181 |
| D6L | VAR/D5L, CPV/C8L | Interleukin 18-binding protein | Y53, L55, F67 |
| E13L | VAR/N3L, VAC/D13L | Needed for immature IMV surface membrane | F168, F486,F487 |
| A22R | VAR/A21R, VAC/A20R | Processivity factor for viral DNA pol | Q146, G203, E204 |
| I7L | VAR/K7L, VAC/I7L | Virion core protein, DNA topoisomeras II homolog | L239, H241, C328 |
| C19L | VAR/C17L, VAC/F13L, CPV/G13L | Envelope protein | C53, N55, S58 |
| A50R | VAR/J4R, VAC/A50R, CPV/A53R | DNA ligase | C11, Y15, C147 |

Table 2 Structure acquisition methods, structural soundness scoring and docking success rate of 12 key proteins

| ORFs | Methods | Protein Category | pLDDT | Rate%* | ORFs | Methods | Protein Category | pLDDT | Rate% |
| --- | --- | --- | --- | --- | --- | --- | --- | --- | --- |
| A49R | RCSB, DS | **VAR**, VAC, CPV, MPV | - | 78.79 | C3L | RCSB, DS | VAR, **VAC**, CPV | - | 82.32 |
| H1L | RCSB, DS | VAR, **VAC**, CPV, MPV | - | 68.19 | E4R | RCSB, DS | VAR, **VAC**, CPV, MPV | - | 77.78 |
| C7L | RCSB, DS | **VAR**, VAC, CPV, MPV | - | 83.33 | A41L | RCSB, DS | **VAR**, VAC, CPV, MPV | - | 73.23 |
| D6L | RCSB, DS | VAR, VAC, CPV, MPV | - | 79.30 | E13L | RCSB, DS | VAR, **VAC**, CPV, MPV | - | 76.26 |
| A22R | AlphaFold2, DS | VAR, VAC, **MPV** | 90.3 | 85.29 | I7L | AlphaFold2, DS | VAR, VAC, CPV, **MPV** | 86.9 | 82.32 |
| C19L | AlphaFold2, DS | VAR, VAC, **MPV** | 91.6 | 75.25 | A50R | AlphaFold2, DS | VAR, VAC, CPV, **MPV** | 91.2 | 69.70 |

*: Proportion of docking results with docking affinity below -0.5 in the small molecule database

**Specific comment** 4

Given that a significant portion of the protein crystal structures remains unresolved, the authors generated homology models to address this limitation. How did the authors predict the orthosteric sites of the protein targets? Was a validation conducted against known inhibitors to assess the accuracy of the predicted orthosteric sites?

**Response** 4

Thanks to the reviewers for their professional advice. Proteins are important functional molecules in living organisms, and their structures determine their functions and interaction modes. Accurate three-dimensional structures of proteins are of key significance in revealing their biological functions, disease mechanisms, and drug development. In our work, the monkeypox virus protein structure was targeted mainly by template-based homology modal bonding and sequence-based modal bonding. Discovery Studio program (DS), as a professional molecular simulation software for life sciences, has been used in the characterization of proteins (including protein-protein interactions), homology modeling, molecular mechanics calculations and molecular dynamics simulations, structure-based drug design tools ( including ligand-protein interactions, novel drug design and molecular docking), small-molecule-based drug design tools (including quantitative conformational relationships, pharmacophore, database screening, ADMET), and design and analysis of combinatorial libraries. By using DS, a reasonable protein structure can be well obtained based on amino acid sequence mode bonds with certain sequence similarity, and this technique is also widely used and verified to be reasonable by major groups. For the sequence similarity content of protein structures, we have also made corresponding additions to demonstrate the high sequence similarity among orthopox viruses and the rationality of obtaining structures through homologous mold bonds. Here we give some literature on the use of the program to die bond protein structures in order to open the work: Studio D. Accelrys, 2008, 420; Evers A, Klebe G. Journal of medicinal chemistry, 2004, 47(22): 5381-5392; Duan H, Hu K, Zheng D, et al. International Journal of Biological Macromolecules, 2022, 223: 1562-1577. For protein structures without templates, we chose the AlphaFold2 technique, which has gained more recognition in recent years. As a deep machine learning method, AlphaFold2 achieves an average accuracy of 92.4 for predicting proteins from scratch, which is almost comparable to experimentally resolved structures. Using the AlphaFold2 technique, we selected the highest scoring structures as rational structures and used them for subsequent drug screening work. In addition, the reviewer asked how to predict the orthosteric site of the protein and whether to verify the site with inhibitors. We thank the reviewers for asking this question again, and we understand that the orthosteric site is the active site that exerts binding to the substrate. In the previous question we explained that all binding sites were obtained experimentally and not by molecular modeling techniques. For the active site validation problem, we also gave a detailed explanation in the third question and corrected the content of the main text. The docking results showed that more than 68% of the compounds in the small molecule library were successfully bound to the correct site, which proves the accuracy of the active site from the side.

**Specific comment** 5

If the authors have not undertaken validation utilizing established inhibitors against the protein targets, I recommend conducting a benchmark study for comprehensive evaluation.

**Response** 5

We thank the reviewers for again asking questions about the validation of protein targets against established inhibitor libraries, and we are concerned that this is a very important issue for the article. In the third question, the reviewer asked for the active site whether reverse molecular docking was performed, and we also added the protein-inhibitor library docking section to the active site confirmation section to validate the protein targets. It was found that each protein was able to bind better (<-0.5) to more than 68% of the small molecules in all 12 systems, demonstrating the soundness of the methodology while proving the correctness of the system binding pocket selection. Here we give a correction to this part of the article:

Table 2 gives the methods for obtaining the 12 key protein structures, the types of modeled protein structures and the scoring results of the AplhaFold2 modeled structures. It can be found that basically all the orthopoxvirus genus proteins can be reasonably molded and the simulated structures are relatively reasonable. Figures s1-s16 show the reasonableness of the modeled structures of each system. Taking the A22R modeling results as an example (Fig. s9, s10), in the AlphaFold2 modeling results, rank_4 has the highest pLDDT and Ptmscore for the five modeled structures given, which means that this structure is the most representative of the dominant conformation of A22R. Specifically, from the Sequence coverage and predicted IDDT, it is easy to find that the residues of the protein are in high Sequence identity and more than 98 residues are in high pIDDT values (pIDDT > 60) [107]. Based on rank_4, we used Discovery Studio to model the VAR, VAC, and MPV, and the results showed that over 95% of the amino acids were in the conformational optimum region, and only a few amino acids (VAR:5, VAC:7, MPV:6) were in the conformational inappropriate region. Considering that the protein contains more than 400 amino acids and the amino acids in the unreasonable region are in the loop region, which is far from the core region of the protein, it is considered reasonable to build the conformation [28, 108]. In addition, further reverse molecular docking work was used to verify the rationality of the protein structure. Through the Autodock vina program package, we docked 189 small molecules to each protein separately. As shown in Table 2, more than 135 (more than 68%) were docked with affinity less than -5 for each system, indicating that the protein active site is relatively reasonable and can ensure the follow-up work.

Table 2 Structure acquisition methods, structural soundness scoring and docking success rate of 12 key proteins

| ORFs | Methods | Protein Category | pLDDT | Rate%* | ORFs | Methods | Protein Category | pLDDT | Rate% |
| --- | --- | --- | --- | --- | --- | --- | --- | --- | --- |
| A49R | RCSB, DS | **VAR**, VAC, CPV, MPV | - | 78.79 | C3L | RCSB, DS | VAR, **VAC**, CPV | - | 82.32 |
| H1L | RCSB, DS | VAR, **VAC**, CPV, MPV | - | 68.19 | E4R | RCSB, DS | VAR, **VAC**, CPV, MPV | - | 77.78 |
| C7L | RCSB, DS | **VAR**, VAC, CPV, MPV | - | 83.33 | A41L | RCSB, DS | **VAR**, VAC, CPV, MPV | - | 73.23 |
| D6L | RCSB, DS | VAR, VAC, CPV, MPV | - | 79.30 | E13L | RCSB, DS | VAR, **VAC**, CPV, MPV | - | 76.26 |
| A22R | AlphaFold2, DS | VAR, VAC, **MPV** | 90.3 | 85.29 | I7L | AlphaFold2, DS | VAR, VAC, CPV, **MPV** | 86.9 | 82.32 |
| C19L | AlphaFold2, DS | VAR, VAC, **MPV** | 91.6 | 75.25 | A50R | AlphaFold2, DS | VAR, VAC, CPV, **MPV** | 91.2 | 69.70 |

*: Proportion of docking results with docking affinity below -0.5 in the small molecule database

**Specific comment** 6

What rationale led the authors to choose AutoDock Vina? Kindly elucidate the justification for this selection. Additionally, is it feasible to conduct reverse screening of peptide-like inhibitors using the web server?

**Response** 6

We thank the reviewers for their questions about the methodology and we are happy to give explanations about the choice of methods. Molecular docking can be used to predict the binding conformation of compound small molecules in biological macromolecules and their binding ability, which plays an important role in the study of protein-ligand interaction mechanisms and structure-based computer-aided drug design. Based on different optimization methods and evaluation functions, common molecular docking programs include Autodock, Dock, Affnity, FlexX, Gold, Hex, and so on. Among them, Autodock, as an open-source and free molecular docking software, adopts Lamarckian genetic algorithm to find the optimal binding position of ligand and receptor, which has the advantages of fast computation speed and accurate algorithm, and it is especially suitable for building virtual screening based on molecular docking. Currently, Autodock includes two generations of products, Autodock4 and Autodock Vina, the former mainly includes two major programs grid and dock, respectively, pre-calculating grid and binding small molecules to target proteins according to grid parameters; While AutoDock Vina does not require choosing atom types and pre-calculating grid maps for them. Instead, it calculates the grids internally, for the atom types that are needed, and it does this. In addition, Autodock Vina is the most commonly used molecular docking software today because of its faster speed and accuracy compared to Autodock4. In summary, considering the software fee, calculation rate, accuracy and market recognition, we finally chose Autodock Vina as the platform docking method. In addition, the reviewer asked whether reverse screening was possible for peptide-like proteins. In our server, in addition to common small molecule inhibitors, peptide inhibitors can also be used as screening substrates, and the operation process is the same as that for small molecule inhibitors.

**Specific comment** 7

The authors must mention how their server is unique/better than similar tools developed in the past, such as the 'COVID-19 Docking Server'.

**Response** 7

In the manuscript, the description of the server does not address the comparison with other servers of the same type, and we thank the reviewers for being able to raise this issue to ensure the completeness of the article. Compared with similar tools developed in the past, the main features in our server include four aspects: a screening platform for orthopoxviral drugs/targets, piggybacking on potential orthopoxviral targets and possible inhibitor libraries, user-friendly interface and binding mode visualization for the results, high computation rate and accuracy, and so on. These features ensure that the server is a target/drug screening platform for monkeypox virus and cannot be used for other disease applications. Finally, the content about the server in our most manuscript has been further revised, and the specific amendments are shown below:

The MPV Docking Server, an online meta-server for key protein target prediction and potential drug screening for MPV, was built using Autodock Vina as the docking engine, targeting MPV as well as other orthopox viruses (VAR, VAC and CPV). Compared to other docking servers of the same type, this server provides a user-friendly interface and binding mode visualization for the results, rendering it a useful tool for orthopox virus drug and key protein discovery. In addition, through extensive literature and database screening, three virtual databases were constructed: MPV open reading frame, orthopox virus key protein library and inhibitor database, which provide theoretical guidance for subsequent development based on MPV and orthopox virus and greatly accelerate the discovery of therapeutic drugs for monkeypox and address the current critical situation.

**Specific comment** 8

In the literature study in the introduction, the authors should also cite one recent exciting work published in ACS JPCL, where the authors modeled the P37 protein of MPXV using the AF tool and performed computer-aided drug discovery. The paper suits the context of the webserver as well the manuscript. <https://doi.org/10.1021/acs.jpclett.3c00087>

**Response** 8

We thank the reviewers for their comments. We downloaded and read the article in detail and found that the work of Rana et al. is indeed more relevant to this paper, and we have taken the reviewers' comments on board and cited the literature in our article.

In recent years, scientists have conducted a lot of research to obtain effective anti- monkeypox virus drugs through bioinformatics analysis, drug database screening, homologous disease drug modification and other methods [16, 4, 5, 6, 8, 9].

[16] Sen Gupta P S, Panda S K, Nayak A K, et al. Identification and investigation of a cryptic binding pocket of the P37 envelope protein of monkeypox virus by molecular dynamics simulations. The Journal of Physical Chemistry Letters, 2023, 14(13): 3230-3235.

**Specific comment** 9

Additionally, the authors are advised to improve the website to make it more user-friendly:

a. The user interface must be made to look interesting and organized. Various visualization tools are now available to present 3D protein structures and interactions. The authors should work on it.

b. The authors should think of a simple one/two-worded name for the server, implying that it is a docking server meant only for MPXV proteins and also serves as a comprehensive database.

c. The manuscript title can also be improved. It does not resonate with the work done or the core feature of the server.

**Response** 9

We thank the reviewers for their suggestions about the website, and we will respond to each of the three issues mentioned.

Firstly, for the user interface, we have optimized the webpage, mainly including the main page directory adjustment, page background color optimization and server name. The reviewer mentioned the visualization tool for 3D protein structure and interactions, which has been loaded into the server. When users submit and complete the tasks through the platform, they can use the visualization tool to view the docking status of the substrate on the protein online, and filter the docking results in a simple way.

Secondly, the reviewer suggested replacing the name of the server with a simple single/two words, considering that the server not only realizes docking work but also contains two databases. We think the reviewer's suggestion is very good and are happy to change the name of the server based on the reviewer's comments. The current server’s name is Monkeypox Virus Docking Server and we have optimized the server’s name to, MPXV-TG finder: a server dedicated to seeking Monkeypox virus targets/drugs.

Finally, regarding the manuscript title issue. This issue was also mentioned several times by the previous reviewer. We thank the reviewer for raising this issue, and we have also adjusted the title of the manuscript based on summarizing the full text, and the new title is entitled: Identification of core therapeutic targets for Monkeypox virus and repurposing potential of drugs against them: an in-silico approach.
