# Supplementary material for: Identification of core therapeutic targets for Monkeypox virus and repurposing potential of drugs: A WEB prediction approach
Source: PLoS One. 2024 Dec 6;19(12):e0303501. doi: 10.1371/journal.pone.0303501 (PMC11623562; doi:10.1371/journal.pone.0303501)
Supplement: Attachment — Submitted filename: Response to Editor.docx [file pone.0303501.s021.docx]

Dear Editorial Office

Editor, ***PLOS ONE***

Thank you very much for your email of Major decision, regarding our manuscript: **Identifying the core therapeutic targets of monkeypox virus and the repurposing potential of orthopox inhibitors: an in-silico approach (Manuscript Number: PONE-D-24-03917R1).**

It's good to hear from you, and we're responding to the questions mentioned by Editor. First of all, our article is duplicated with another article title. When the reviewer gave his comments, he suggested us to change the title several times and gave us this title. At that time, we thought the title was suitable for our article and applied it, forgetting to check whether it was a duplicate of another article's title. We will revise the title of the article appropriately. Secondly, the editor mentioned that the article has similarity in content. Both articles were used as the core therapeutic target of monkeypox protein, and it is necessary to understand the story behind monkeypox. In addition, the focus of the two articles is different, our article mainly focuses on the important targets and potential inhibitors of monkeypox to screen possible drugs and speculate other targets, while the other article focuses on the connection between specific targets and inhibitors. The originality of the article is a must for every researcher, and we guarantee that there is no plagiarism or falsification in the writing and experimental process of this article.

We look forward to receiving your revised manuscript.

Kind regards,

Dr. Hu
